# Microbial bioleaching of rare earth elements from phosphate minerals: a biotechnology-driven systematic review of mechanisms, bioprocess determinants, and opportunities for sustainable recovery

**DOI:** 10.1186/s12896-026-01125-1

**Published:** 2026-03-03

**Authors:** Sonali Prabodha Vijayarathna, Ileperumaarachchige Vayanga Nishani Rathnayake, Pradeep Wishwanath Samarasekere

**Affiliations:** 1 Center for Advanced Materials and Smart Manufacturing, Faculty of Computing and Technology, University of Kelaniya, Kelaniya, 11600 Sri Lanka; 2 Department of Microbiology, Faculty of Science, University of Kelaniya, Kelaniya, 11600 Sri Lanka

**Keywords:** REE recovery, Monazite, Apatite, Fungal bioleaching, Bacterial bioleaching, Organic acid production, Phosphatase activity, Bioprocess optimization, Biofilm-mediated dissolution, Non-sterile systems, Leaching kinetics, Sustainable extraction

## Abstract

**Background:**

Rising demand for rare earth elements (REEs) and the severe environmental impact of conventional extraction from phosphate minerals (monazite, apatite) have intensified the search for green alternatives. Microbial bioleaching offers a low-energy, low-waste, and a sustainable biotechnological alternative by exploiting the ability of fungi and bacteria to generate organic acids, siderophores, reducing agents, and other metabolites that solubilize REEs. Although interest in REE bioleaching has increased, a biotechnology-focused synthesis of microbial mechanisms, metabolic constraints, and process determinants specific to phosphate matrices remains limited.

**Methods:**

A PRISMA-guided systematic review was conducted. Scopus, Web of Science, PubMed, and Google Scholar and other major databases were searched to identify peer-reviewed studies reporting microbial bioleaching of REEs from phosphate minerals. From 443 identified records, 25 studies met the inclusion criteria after screening and eligibility assessment. These studies were evaluated based on microbial species, metabolic mechanisms, culture conditions, mineral substrates, and REE solubilization performance.

**Results:**

Fungal species, particularly *Aspergillus*, *Penicillium* and *Paecilomyces* demonstrated the highest REE mobilization efficiencies through intensive production of citric, oxalic, and gluconic acids, along with phosphatase activity. Bacterial strains, including *Acidithiobacillus*, *Bacillus*, *Pantoea*, *Burkholderia*, *Pseudomonas*, and *Klebsiella* contributed complementary mechanisms such as proton extrusion, siderophore secretion, and Fe(III) / Fe(II) redox cycling. Bioleaching performance was strongly influenced by media composition, carbon source, nitrogen assimilation, pH evolution, mineralogy of the phosphate substrate, pulp density, and particle size. Across studies, the lack of standardized conditions limited direct comparability, but organic acid dominated pathways consistently produced the most robust REE solubilization.

**Conclusions:**

Microbial bioleaching is a promising biotechnological platform for REE recovery from phosphate minerals, driven by metabolically diverse acidogenic, chelating, enzymatic, and redox mechanisms. However, advancements remain constrained by heterogeneous methodologies, limited integration of mechanistic studies, and minimal use of engineered strains or controlled bioreactor systems. Future progress requires standardized experimental frameworks, improved mechanistic understanding of organism-specific roles, and rational design of optimized microbial systems. This review offers a biotechnology-centered foundation to guide next-generation research on sustainable REE mobilization from phosphate resources.

**Supplementary Information:**

The online version contains supplementary material available at 10.1186/s12896-026-01125-1.

## Background

Rare earth elements (REEs) are indispensable for modern technologies ranging from permanent magnets, electric vehicles, wind turbines, and lasers to catalysts, semiconductor devices, and advanced medical imaging systems [1–3]. REEs comprise the fifteen lanthanides together with yttrium and scandium, several of which are identified as critical raw materials due to supply vulnerability [4, 5]. Securing sustainable and environmentally responsible routes for REE extraction has become a global priority as the global demand accelerates [6–8]. Conventional processing of REE bearing phosphate minerals, such as monazite, apatite, and related phosphate rock, typically involves high temperature cracking, concentrated mineral acids, and aggressive chemical oxidants [9–12]. These methods generate hazardous waste streams, require substantial energy input, and present significant occupational and environmental risks. The natural occurrence of REEs in phosphate matrices, formed through geological phosphate precipitation processes and common in igneous phosphorites, placer deposits, and mineral sands, further complicates their extraction [13–15]. The need for cleaner extraction strategies has thus intensified research interest in biotechnological alternatives capable of mobilizing REEs under milder and more eco compatible conditions [16–20].

Microbial bioleaching offers a promising path forward by exploiting the natural metabolic capacity of microorganisms to solubilize metals through organic acid generation, proton extrusion, siderophore secretion, redox reactions, and complexation processes [21–25]. Fungi such as *Aspergillus*, *Penicillium*, and *Paecilomyces* are well-known for their high yield organic acid production, especially citric, gluconic, and oxalic acids, which effectively attack phosphate mineral lattices [26–28]. Bacterial genera including *Acidithiobacillus*, *Bacillus*, *Burkholderia*, *Enterobacter*, *Klebsiella*, *Microbacterium*, *Pantoea*, *Pseudomonas*, *Streptomyces*, etc. contribute additional mechanisms such as iron cycling, biosurfactant production, and ligand mediated chelation [29–31]. These phosphate solubilizing microorganisms (PSMs) play a central role because phosphate dissolution, mediated by organic acids, chelators, and phosphatases directly enhances REE release from the phosphate matrix. Phosphorus solubilization is also vital for microbial cellular metabolism and energy transfer, influencing overall bioleaching activity. When applied to REE-bearing phosphates, these microbial activities collectively facilitate the dissolution of the mineral matrix and promote the release of REEs into solution.

Unlike sulfidic ore bioleaching, which is primarily driven by iron and sulfur oxidation reactions generating ferric iron and sulfuric acid as chemical oxidants, phosphate mineral bioleaching relies predominantly on acidolysis and ligand-mediated complexation to disrupt REE-phosphate bonds. Sulfide systems are governed by redox cycling mechanisms, whereas phosphate matrices lack redox-active metal centers and therefore require proton attack and chelation for dissolution. This mechanistic distinction necessitates tailored microbial strategies and medium design when transitioning from sulfide-based biomining paradigms to phosphate-hosted REE systems [32–36].

Despite increasing interest in REE bioleaching, progress has been hindered by fragmented investigations, variations in microbial systems, and substantial differences in mineralogical substrates. Much of the current literature remains descriptive, with limited integration of biotechnology concepts such as metabolic engineering, pathway regulation, or omics driven strain characterization.

A systematic, biotechnology focused synthesis is therefore needed to deepen understanding of microbial mechanisms and identify the parameters most critical to bioleaching success. This review addresses this gap by evaluating published studies on microbial REE bioleaching from phosphate based minerals, with emphasis on: (i) microbial species and metabolic pathways involved, (ii) culture conditions that modulate bioleaching performance, (iii) substrate specific responses, and (iv) opportunities for engineering more efficient biological systems. By framing REE bioleaching as an emerging bioprocess technology rather than a purely mineral processing technique, this review provides a comprehensive foundation for advancing microbial strategies toward scalable, sustainable REE recovery.

## Methods

This systematic review was conducted following the Preferred Reporting Items for Systematic Reviews and Meta-Analyses (PRISMA) guidelines [37, 38]. Specifically, the PRISMA extension for systematic reviews was used to structure the identification, screening, eligibility, and inclusion phases. A comprehensive database search of Scopus, Web of Science Core Collection, PubMed, and Google Scholar, and publisher databases including Elsevier, Taylor and Francis, MDPI, and Springer was performed to identify peer-reviewed studies reporting microbial bioleaching of rare earth elements from phosphate minerals. The keywords employed for the search encompassed “Bioleaching”, “Rare Earth Elements”, “Microorganisms”, “Primary sources”, and “Phosphate minerals”. Boolean keyword combinations such as “rare earth elements” AND “bioleaching”, “REE” AND “microorganisms”, “rare earth elements” AND “Primary sources” AND “bioleaching” etc. were used to ensure broad coverage of the literature. Searches were conducted from March 2023 to August 2025 to reflect the updated literature base. No restrictions were placed on publication date to capture both foundational and recent studies.

Studies were considered eligible if they employed microorganisms, including fungi, bacteria, or mixed cultures to leach REEs from phosphate bearing minerals such as monazite, apatite, phosphate rock, or monazite rich tailings. Eligible studies were required to quantify REE mobilization or mineral dissolution through analytical methods and to report experimental conditions in sufficient detail to enable interpretation. Inclusion criteria additionally encompassed studies focused on bioleaching, bio-recovery, biomining of REEs, or microbial leaching from primary phosphate sources. Articles focusing solely on chemical leaching, studies using synthetic REE salts instead of mineral substrates, non-peer reviewed sources such as theses, book chapters, conference abstracts, or conceptual reviews without original data were excluded. Studies involving bioleaching of REEs from secondary sources such as waste materials, red mud, or coal ash were also excluded to maintain a strict focus on primary phosphate minerals.

The eligibility step involved a thorough examination of key parameters. Two independent reviewers conducted title, abstract, and full-text screening. Conflicts were resolved by consensus, and disagreements that remained unresolved were adjudicated by a third reviewer. This process ensured consistent application of eligibility criteria and reproducible selection of studies.

**Fig. 1:**
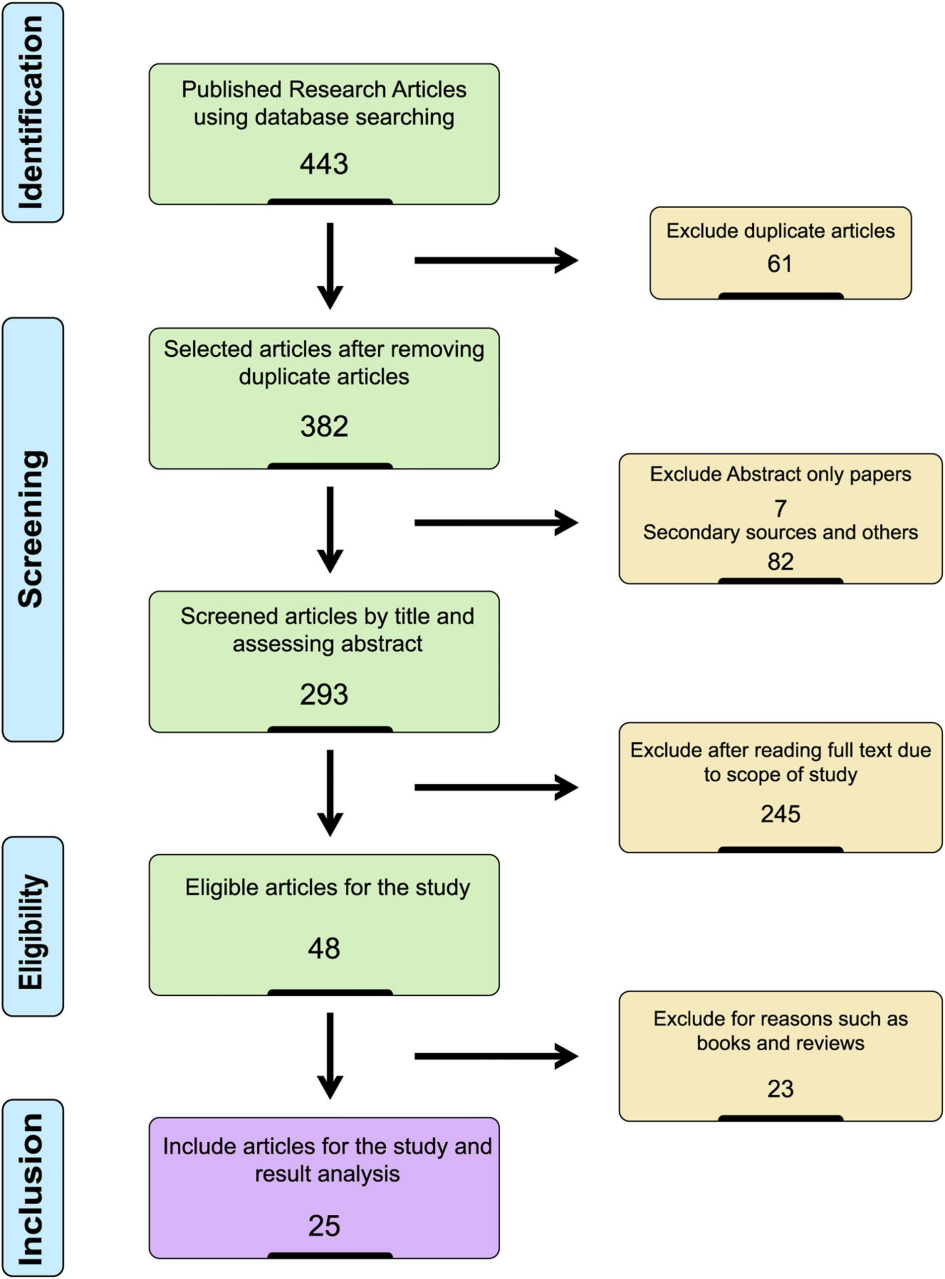
PRISMA Flow diagram explaining the search strategy, inclusion and exclusion criteria used for the systematic review of scientific literature

The titles and abstracts of all retrieved articles were screened, and full texts of potentially relevant papers were examined in detail. A total of 443 records were identified, 61 duplicates were removed, and 382 articles were screened. After removing 89 records during title and abstract screening, 293 full-text articles were assessed for eligibility. Of these, 245 were excluded for not meeting inclusion criteria, along with six book chapters and seventeen review articles. Twenty-five studies met all criteria and were included in the final synthesis. The overall screening and selection process is illustrated in the Fig. 1 as PRISMA flow diagram.

Data extraction was performed manually by two independent reviewers using a predefined structured template. Study quality was assessed descriptively. The extracted variables included microbial strain identity, mineral substrate type, culture conditions, leaching configuration (direct vs. indirect), reported REE concentrations or recovery percentages, and proposed mechanistic interpretations. Cross-checking was conducted through independent comparison of extracted datasets, and discrepancies were resolved by consensus discussion. No automated text-mining or AI-assisted extraction tools were used in the systematic analysis. All eligible studies were included in the synthesis regardless of quality scores, although study limitations were considered when interpreting results.

A decision was made against conducting a quantitative meta-analysis as the included studies exhibited high methodological heterogeneity, including variations in microbial strains, substrate compositions, pulp densities, incubation periods, and reporting units (e.g., mg/L vs. percentage recovery). This precluded reliable pooling of data. Attempting meta-analysis under such heterogeneity would risk producing misleading aggregated values. Instead, a qualitative synthesis was prioritized to identify overarching trends and biotechnological insights focusing on microbial mechanisms, performance trends, and biotechnological determinants across the twenty-five included studies, while acknowledging these variabilities.

## Results

### Microbial mechanisms supporting REE bioleaching from phosphate minerals

**Fig. 2:**
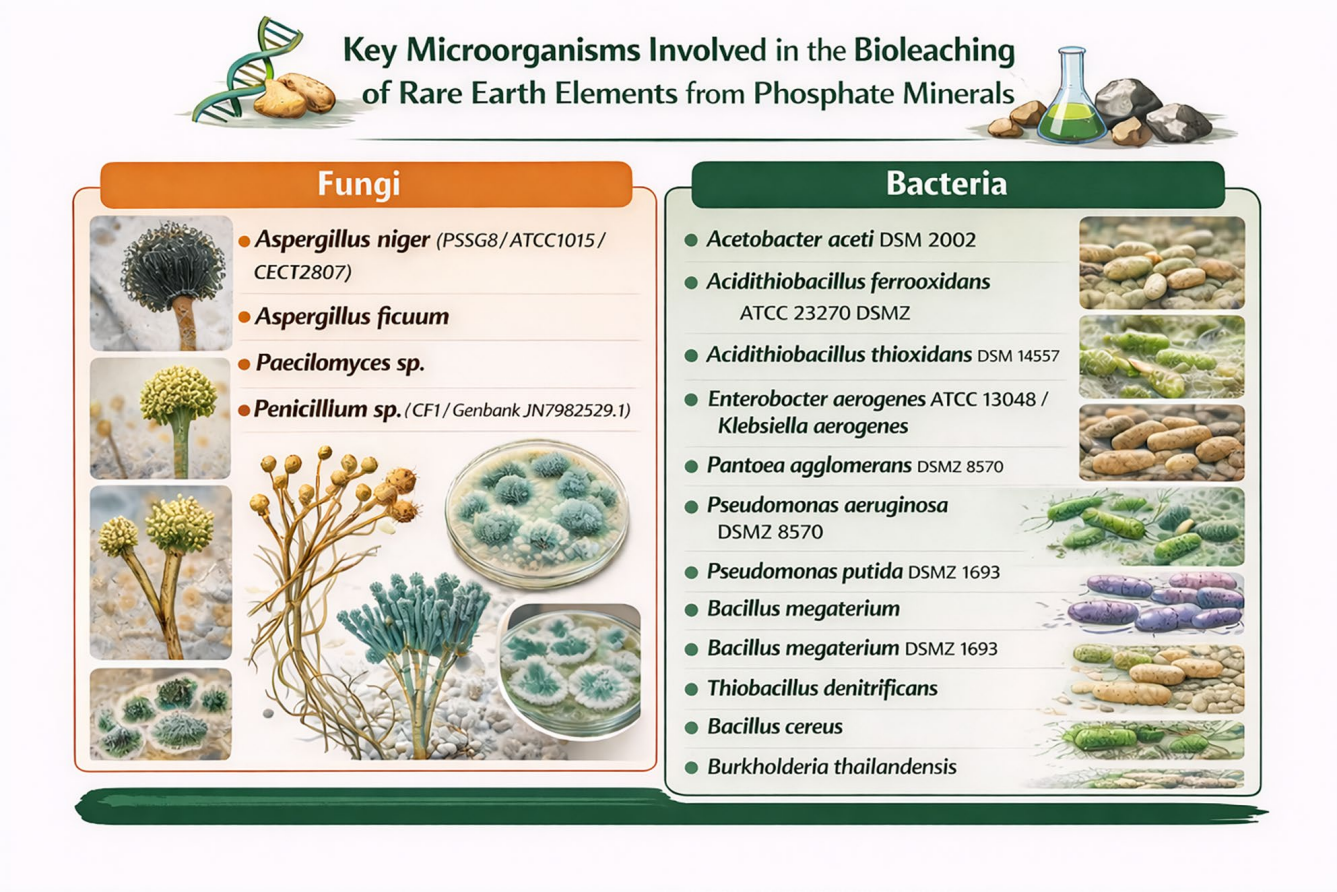
Reported microorganisms for bioleaching of rare earth elements from phosphate minerals

The twenty-five studies included in this review collectively demonstrate that microbial bioleaching of rare earth elements from phosphate minerals is driven by a diverse set of biochemical and physiological mechanisms [39–60, 64–66]. These mechanisms operate either independently or synergistically, and their dominance varies according to microbial species, nutrient availability, and mineralogical characteristics of the substrate. A diverse range of bacterial and fungal species were employed across the studies (Table 1; Fig. 2). Across all studies, four mechanistic categories emerged as central to REE solubilization: (i) organic acid-mediated acidolysis, (ii) proton extrusion and acidification, (iii) siderophore and ligand assisted complexation, and (iv) redox induced structural destabilization. Recent work further demonstrates the contributions of rhamnolipid biosurfactants [58], and biological pretreatment of carbonatite ores [59, 60], expanding the mechanistic toolbox beyond traditional organic acid production (see Table 2).

#### Organic acid-mediated dissolution as the primary mechanism

Organic acid production by filamentous fungi was consistently identified as the most potent driver of REE release from phosphate substrates. Species such as *Aspergillus niger*, *Penicillium simplicissimum*, and *Paecilomyces* sp. produced substantial concentrations of citric, oxalic, gluconic, and malic acids, which lowered the pH to values between 1.5 and 3.0 under optimized culture conditions [47, 61, 62]. Citric and oxalic acids in particular promoted strong chelation of REE^3+^, Th^4+^ and Ca^2+^ ions, thereby destabilizing the phosphate lattice. Chelation driven removal of Ca^2+^ and concomitant proton attack on P-O bonds facilitated dissolution of monazite and apatite structures [48, 63]. Across the reviewed studies, fungal systems consistently achieved higher REE solubilization efficiencies compared with bacteria, supporting the central role of acidogenic metabolism in phosphate mineral breakdown. Fungal mycelial morphology affected by temperature, nutrient ratios, and growth phase modulates the interaction between hyphae and mineral surfaces. Temperature-dependent changes in hyphal elongation, branching patterns, and sporulation were shown to contribute indirectly to REE dissolution by altering acid secretion dynamics and surface colonization behavior [45, 46, 55] .

**Table 1:** Microorganisms used in bioleaching process of REE in phosphate mineral

Microorganisms type	Species	Reference
Fungus	*Aspergillus niger /* PSSG8 / ATCC1015 / CECT2807	[39, 41, 43, 44, 47, 53, 54, 56, 64]
	*Aspergillus ficuum*	[40]
	*Paecilomyces* sp WE3F	[43, 55]
	*Penicillium* sp.*/* CF1/ Genbank JN7982529.1	[39, 45, 46, 48]
Bacteria	*Acetobacter aceti* DSM 2002	[42]
	*Acidithiobacillus ferrooxidans/* ATCC 23270 DSMZ	[47, 50, 51, 52, 57],
	*Acidithiobacillus thiooxidans* DSM 14557	[52]
	*Enterobacter aerogenes* ATCC 13048/ *Klebsiella aerogenes*	[46, 49, 50, 51, 65]
	*Pantoea agglomerans* DSMZ 8570	[46]
	*Pseudomonas aeruginosa*	[40]
	*Pseudomonas fluorescence*	[39]
	*Pseudomonas putida* DSMZ 1693	[46]
	*Pantoea agglomerans*	[66]
	*Bacillus megaterium*	[66]
	*Thiobacillus denitrificans*	[59, 60]
	*Bacillus cereus*	[60]
	*Burkholderia thailandensis*	[58]

#### Proton extrusion and medium acidification by bacteria

Although bacterial organic acid production was generally lower than that of fungi, several bacterial strains contributed to REE solubilization through proton extrusion during autotrophic or heterotrophic growth [49, 52, 57]. *Acidithiobacillus ferrooxidans* and *Acidithiobacillus thiooxidans* acidified media through metal oxidation, indirectly enhancing the dissolution of phosphate minerals [21, 67]. Heterotrophic bacteria such as *Bacillus* spp. and *Pseudomonas* spp. lowered pH through metabolic release of protons associated with carbon source oxidation [68, 69]. The magnitude of pH decline in bacterial cultures was generally less pronounced than in fungal systems. However, their contribution became more significant when combined with additional chelating or redox active mechanisms [70, 71].

#### Siderophore and ligand mediated REE complexation

Several bacterial strains, particularly *Pseudomonas* spp. and *Bacillus* spp., produced siderophores and low molecular weight ligands capable of binding REE ions [40, 52, 72, 73]. Although siderophores typically target Fe^3+^, their functional groups (catecholates, hydroxamates, and carboxylates) exhibit affinity toward REE^3+^ cations [74]. Ligand assisted mobilization was often observed in studies where pH reduction alone could not fully account for the extent of REE dissolution. Siderophore associated mobilization was particularly relevant for substrates containing mixed iron and phosphate phases, where microbial chelators facilitated disruption of the mineral surface and enhanced accessibility of REE phosphate bonds.

#### Redox reactions supporting mineral destabilization

**Fig. 3:**
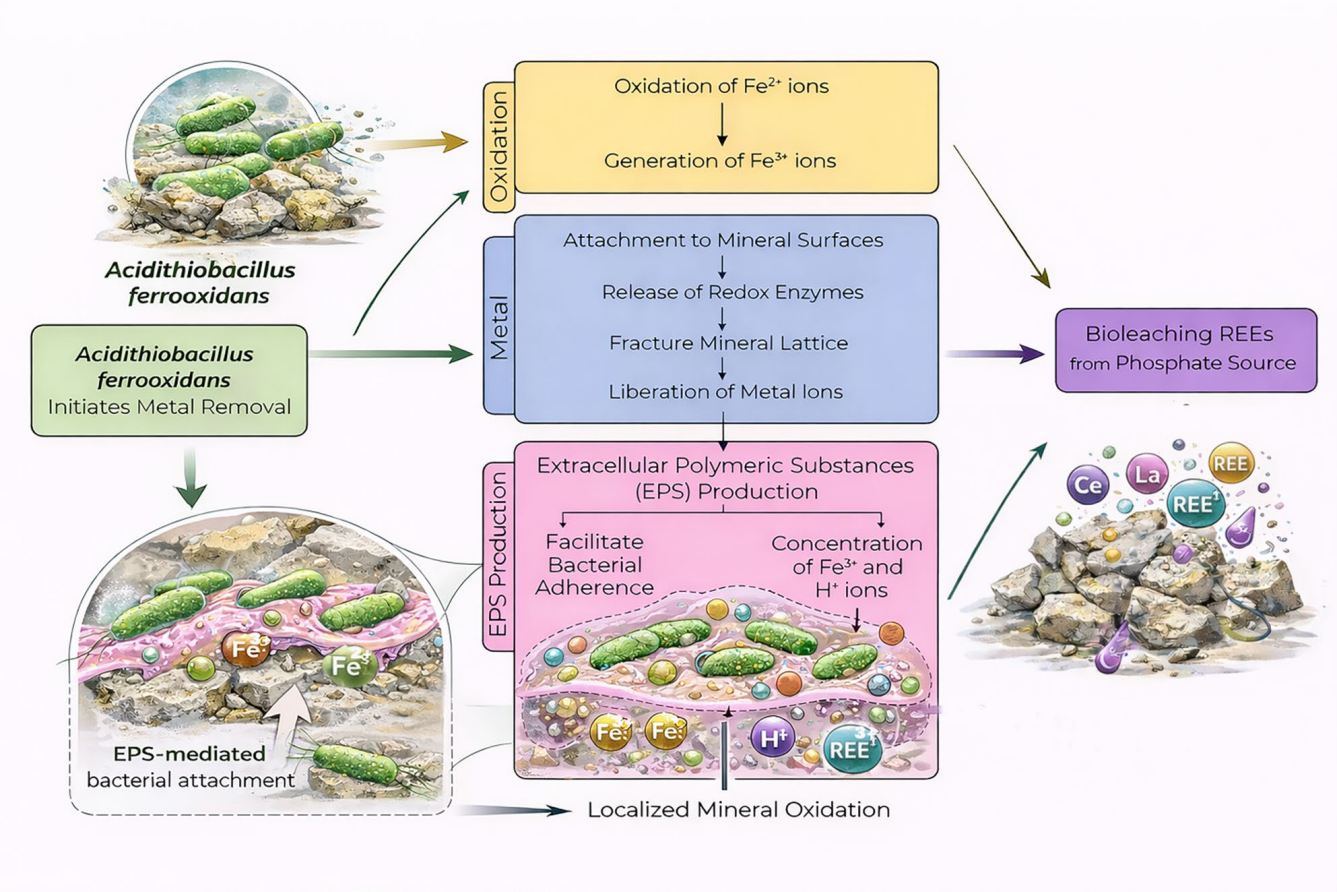
Mechanistic schematic of *Acidithiobacillus ferrooxidans* mediated bioleaching of rare earth elements from phosphate minerals, highlighting Fe^2+^ oxidation, extracellular polymeric substances (EPS) mediated bacterial attachment, localized accumulation of Fe^3+^and H^+^ ions, and enhanced mineral oxidation leading to REE release

Redox interactions were reported primarily in systems involving iron bearing monazite or composite phosphate ores. Autotrophic bacteria such as *Acidithiobacillus ferrooxidans* oxidizes Fe^2+^ to Fe^3+^, generating strong oxidizing conditions that promote mineral dissolution. Initial attachment of *Acidithiobacillus ferrooxidans* to the phosphate mineral surface enables the release of redox-active enzymes, leading to fracture of the mineral lattice and liberation of metal ions. Concurrently, the production of extracellular polymeric substances (EPS) facilitates bacterial adhesion and forms a localized microenvironment at the mineral-bacteria interface. The EPS matrix promotes accumulation of Fe^3+^ and H^+^ ions, enhancing localized mineral oxidation and accelerating the release of REEs from the phosphate matrix as illustrated in Fig. 3 [47, 50, 57]. Although REE cations themselves do not undergo redox cycling, redox-driven alterations of surrounding mineral phases enhanced structural weakening and promoted dissolution. These reactions were less dominant than organic acid pathways but played a complementary role in multi-step or mixed culture systems.

#### Biofilm formation and mineral dissolution

In addition to planktonic metabolic activity, a subset of the reviewed studies reported that surface-attached microbial growth and biofilm formation enhance REE mobilization from phosphate minerals. Biofilms consist of structured microbial communities embedded within extracellular polymeric substances that facilitate close and sustained contact between cells and mineral substrates. This spatial proximity enables localized physicochemical conditions at the microbe-mineral interface that differ from bulk solution chemistry. Tian et al. demonstrated that *Acidithiobacillus ferrooxidans* attaches to phosphate rock surfaces during REE bioleaching, with iron oxidation occurring within the EPS matrix. Proton generation and ferric iron accumulation at the cell-mineral interface contributed to localized destabilization of the phosphate matrix, resulting in enhanced REE release relative to what would be expected from bulk acidity alone [57]. Similarly, Fathollahzadeh et al. observed greater REE mobilization from monazite when direct microbial contact was permitted compared to systems where mineral cell interaction was restricted [49], supporting a contact-mediated dissolution mechanism. Feng et al. reported that bacterial dissolution rates of fluorapatite exceeded predictions based solely on solution-phase chemical equilibria [66]. Although the study focused primarily on phosphate release, the mineralogical relevance to REE-bearing phosphate systems suggests that surface-associated microbial activity contributes to enhanced mineral breakdown. Thus, the biofilm-mediated processes may create microenvironments that promote ligand complexation, proton retention, and redox reactions at the mineral surface, thereby increasing dissolution efficiency beyond indirect metabolite-driven leaching alone.

### Fungal systems

Fungal species consistently emerged as the most effective microbial agents for mobilizing REEs from phosphate minerals across the studies included in this review. Their superior performance is primarily attributed to the exceptional capacity to synthesize and secrete high concentrations of low molecular weight organic acids, which create acidic microenvironments and provide powerful chelating ligands for REE solubilization. Filamentous fungi such as *Aspergillus niger*, *Aspergillus terreus*, *Aspergillus ficuum*, *Penicillium simplicissimum*, *Penicillium citrinum*, and *Paecilomyces* sp. were repeatedly shown to generate organic acid profiles dominated by citric, oxalic, gluconic, and malic acids [43–45, 48, 54]. These metabolites not only lower the medium pH but also form stable complexes with REE^3+^ ions, facilitating detachment of structural cations and destabilization of phosphate lattices such as monazite and apatite.

Several studies highlighted particularly strong bioleaching performance from fungal isolates capable of producing diverse and abundant organic acids. High performance strains including *Paecilomyces* WE3-F and *Aspergillus terreus* ML3-1, reported by Brisson et al., achieved some of the highest dissolved REE concentrations among all reviewed studies, reaching approximately 112 mg L^− 1^ and 101 mg L^− 1^, respectively, under optimized culture conditions [43]. These findings underscore the significance of fungal metabolic plasticity, particularly the capacity to shift between different acidogenic pathways depending on medium composition and nutrient availability. *Aspergillus niger*, one of the most extensively studied fungal bioleaching agents, also achieved notable REE release from monazite bearing materials with concentrations recorded in the range of 0.701 mg L^− 1^ to 86 ± 6 mg L^− 1^ at early or mid-incubation points depending on medium composition [41, 43]. Other studies, including those by Keekan et al. and Castro et al., demonstrated that *Aspergillus niger* can effectively solubilize cerium and mixed REEs from monazite bearing minerals, with measurable release occurring within the first week of incubation and influenced strongly by carbon source type and concentration [44, 54]. *Aspergillus ficuum* in another study achieved extraction levels approaching 75%, reflecting strong acidogenic potential and well aligned substrate properties [40]. *Penicillium* species, including *Penicillium simplicissimum* and *Penicillium citrinum* displayed both effective organic acid production and phosphatase activity, producing mid-range REE concentrations that exceeded those of most bacterial systems under similar conditions. In addition to organic acid secretion, some fungi exhibit extracellular phosphatase activity that contributes to mineral breakdown. Corbett et al. reported that *Penicillium* sp. CF1 with measurable cellular and extracellular acid phosphatase activity together with effective REE release monazite systems [48]. However, phosphatase production occurred at the same time as organic acid-driven acidification and surface-associated microbial activity. Phosphatase activity alone was insufficient to explain the level of leaching that was observed. These results suggest that phosphatase-driven phosphate hydrolysis may support the breakdown of the phosphate structure, but it acts together with other processes. These may include organic acid-mediated dissolution, extracellular polymeric substance formation, and cooperative interactions with native microbial communities. As a result, the improved REE release seen with *Penicillium* sp. CF1 reflects the combined effect of several biochemical and surface-related processes rather than a single dominant enzyme pathway [48]. This highlights the benefit of fungal strains that use multiple modes of mineral transformation in bioleaching systems.

Despite their generally superior performance, fungal bioleaching outcomes varied depending on culture medium, carbon source, mineral substrate composition, and incubation period. Media that enhanced organic acid biosynthesis, such as modified ammonium salts (AMS) or Bromfield medium, tended to produce the most consistent REE release. Conversely, media that restricted acidogenic metabolism or buffered pH, reduced fungal efficiency. These observations reinforce the importance of bioprocess optimization, particularly through medium engineering, and controlled carbon to nitrogen ratios, to maximize fungal bioleaching potential [41, 44]. Across the reviewed fungal studies, both direct contact bioleaching and indirect (spent medium) approaches were employed. Two-step systems, in which organic acids were accumulated prior to mineral exposure, frequently demonstrated enhanced dissolution relative to one-step inoculation, underscoring the importance of metabolite concentration dynamics (see Table 2).

The reviewed evidence establishes filamentous fungi as metabolically versatile and highly effective bioleaching agents for REE-bearing phosphates. Their innate ability to generate abundant chelating acids, coupled in some cases with complementary enzymatic mechanisms, position them as central candidates for developing biotechnology based REE extraction processes.

### Bacterial systems

Bacterial systems contributed a mechanistically diverse but generally more variable set of bioleaching outcomes compared with filamentous fungi. Although bacteria typically produced lower concentrations of organic acids, they demonstrated several distinct biochemical strategies that supported REE mobilization from phosphate minerals. These strategies included proton extrusion during autotrophic or heterotrophic growth, secretion of siderophores and other low molecular weight chelators, and redox cycling of iron in mixed phosphate iron mineral matrices. The relative importance of each mechanism depends on bacterial physiology, nutrient availability, and mineralogical characteristics of the substrate.

Autotrophic acidophiles such as *Acidithiobacillus ferrooxidans* and *Acidithiobacillus thiooxidans* generated acidic environments indirectly through the oxidation of ferrous iron or reduced sulfur compounds. Although the acids produced in these systems were not complex organic acids, the metabolic release of protons combined with the formation of ferric iron (Fe^3+^), contributed to mineral surface attack and structural disruption of REE bearing phases [47, 50, 57]. These redox induced changes also enhanced accessibility of phosphate bonds associated with monazite and apatite. Studies involving *Acidithiobacillus ferrooxidans*, particularly when combined with heterotrophic phosphate solubilizing bacteria, showed that redox dynamics could significantly augment dissolution kinetics suggesting that iron transforming bacteria may play supportive roles in mixed microbial communities [22, 57].

Heterotrophic bacteria such as *Pseudomonas*, *Bacillus*, *Pantoea*, *Enterobacter*, and *Acetobacter* species mobilized REEs primarily through the secretion of chelating metabolites and limited pH reduction associated with carbon metabolism. *Pseudomonas* and *Bacillus* were frequently reported for producing siderophores, catecholate, hydroxamate, or mixed-type compounds that can form stable complexes with trivalent metal ions, including REEs. Although siderophores display the strongest affinity for Fe^3+^, several studies suggested that they may contribute to REE mobilization by destabilizing iron associated mineral phases or by participating in secondary ligand exchange reactions that release REE ions from phosphate matrices [46, 59, 65]. *Acetobacter aceti*, for example, showed comparatively high efficiency among bacterial isolates tested in some studies, likely due to its acidifying metabolism and production of gluconic acid [42].

Compared with fungi, bacterial systems exhibited more modest overall REE dissolution, but their contributions became particularly notable in co-culture or sequential bioleaching configurations. In combined systems evaluated by Fathollahzadeh et al. and others, pairing *Acidithiobacillus ferrooxidans* with heterotrophic phosphate solubilizing bacteria such as *Enterobacter aerogenes* enhanced REE release relative to either organism alone [50]. These cooperative effects were attributed to the simultaneous generation of organic acids, proton driven acidification, and iron redox cycling, which collectively broadened the spectrum of mineral dissolution mechanisms. Certain heterotrophic bacteria, including *Pseudomonas* and *Acetobacter* sp., also achieved relatively higher performance when medium conditions favored siderophore or gluconic acid production. Bacteria also reduced the risk of REE re-precipitation in some mixed systems by producing ligands that stabilized dissolved REE ions in solution.

The reviewed studies indicate that bacteria play an important but often complementary role in REE bioleaching of phosphate minerals. While bacterial systems rarely match the highest dissolution levels achieved by filamentous fungi, their mechanistic diversity and compatibility with fungi in mixed cultures highlight their potential as integral components of engineered consortia or sequential bioprocessing strategies. Their contribution is especially valuable in substrates containing iron-bearing phases or in systems designed to exploit synergistic interactions between acidification, chelation, and redox chemistry. Bacterial investigations included both direct mineral contact systems and indirect leaching using cell-free supernatants. In several cases, indirect systems clarified the relative contribution of secreted metabolites versus cell-associated redox processes, although comparative kinetic data remain limited (see Table 2).

### Influence of culture conditions

Culture conditions exerted a decisive influence on bioleaching performance across all studies, highlighting the importance of bioprocess optimization for effective microbial REE mobilization (see Table 2). Medium composition, carbon and nitrogen sources, pH, temperature, pulp density, particle size, and culture mode were repeatedly identified as primary determinants of microbial metabolite production, the extent, and the rate of phosphate mineral dissolution.

**Fig. 4:**
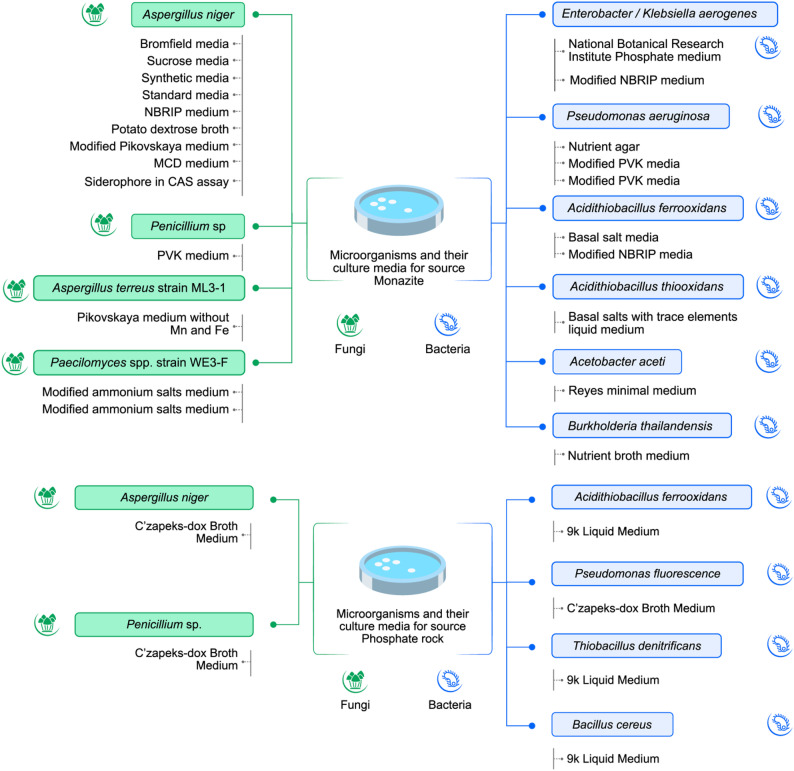
Different microorganisms and the culture media used for monazite, phosphate fluorapatite and phosphorites

Medium formulation was particularly important because it shaped both the quantity and profile of metabolites produced by microorganisms. As illustrated in Fig. 4, different medium compositions were used across these twenty-five studies. Key factors included carbon sources (e.g., glucose, sucrose), nitrogen sources, and phosphorus availability, which is vital for DNA, RNA, ATP, and cell membrane formation of the organisms [75]. Acidogenic fungi responded strongly to carbon rich media, with glucose, sucrose, and starch derivatives stimulating the production of citric, oxalic, and gluconic acids depending on the species. Modified AMS medium [76], Bromfield medium [77], and National Botanical Research Institute’s phosphate growth (NBRIP) medium [78], formulations were among those that yielded high organic acid concentrates in fungal systems, leading to correspondingly higher dissolved REE concentrations. Some studies demonstrated that subtle changes in medium composition, such as replacing glucose with sucrose or adjusting phosphate buffering capacity, altered metabolic pathway fluxes and the acid composition produced. In bacterial systems, medium components influenced siderophore production and iron redox activity with certain nitrogen sources promoting stronger chelator secretion or proton extrusion [39, 41, 47, 50].

Initial pH and its evolution during incubation were critical factors. Although both fungi and bacteria acidified their environment, the rate and the extent of pH decline differed markedly among organisms, and directly governed the dissolution kinetics of phosphate minerals. Fungal strains generally produced faster and more pronounced acidification than bacteria, reaching pH values conducive to REE organic acid complexation within the first few days of incubation. Some bacterial systems, particularly those driven by *Acidithiobacillus* spp., relied on sulfur or iron oxidation pathways to gradually lower pH, resulting in slower but sustained leaching under acidic conditions. Studies consistently showed that lower final pH correlated with increased REE dissolution, but substrate buffering capacity often modulated this relationship [50, 51].

Temperature influenced microbial metabolism, thereby indirectly affecting leaching efficiency. It also influenced fungal morphology, affecting hyphal branching and surface attachment, which indirectly impacted REE dissolution. Although few studies systematically optimized temperature, those that did reported optima aligned with organism physiology. Studies showed that fungal systems generally perform optimally at 25–30 °C which is consistent with the temperature optima for acidogenic metabolism. Some bacterial strains such as *Aspergillus ficuum* and *Pseudomonas aeruginosa* demonstrated temperature optima around 30–35 °C. Mycelial morphology in filamentous fungi, including branching density and hyphal elongation, was shown to be temperature-dependent, suggesting that temperature modulates mineral-biomass contact and therefore dissolution efficiency. Bacterial systems displayed broader thermal tolerance, though fewer studies systematically evaluated temperature dependencies. In most datasets, temperature effects were modest compared with those of medium composition and pH, indicating that metabolic pathway activation was the main driver of performance rather than thermal response [40, 41, 44].

Pulp density and mineral particle size introduced additional constraints. Lower pulp densities typically enhanced dissolution due to improved microbial access and reduced buffering of acids by mineral surfaces, whereas higher pulp densities limited metabolite efficacy and impeded REE release. Smaller particle sizes increased reactive surface area and promoted faster extraction, but excessively fine particles occasionally impaired microbial growth or oxygen transfer. One study reported optimal results at particle sizes around 75 μm within a range of 32 μm to 105 μm and moderate pulp densities, suggesting that physical parameters must be balanced to support both biomass development and mineral reactivity [47]. This observation could be attributed to the reduced surface area and a lower count of active microbial sites in larger particles, hindering the dissolution. In contrast, fine fractions may induce oxygen deficiencies due to reduced airflow rates, potentially resulting in cell damage and deactivation. Further, the particle size reduction could increase particle-particle collisions, leading to significant attrition that might disrupt the cell structures [79, 80].

The mode of culture operation had significant implications for bioleaching efficiency. Two step processes, wherein microorganisms were initially cultivated to accumulate metabolites prior to the introduction of the mineral substrate, consistently outperformed direct one-step inoculations. This improvement was most pronounced in fungal systems, where decoupling growth from mineral exposure allowed cells to reach high metabolic productivity before encountering potentially inhibitory substrates. Mixed culture and co-culture systems also benefited from staged approaches, enabling synergy between metabolic mechanisms such as organic acid production, siderophore release, and iron oxidation [43, 45, 48, 50].

Several studies indicated that REE solubilization increased when acidic metabolites, chelators, and redox active species acted in combination. Two-step bioleaching approaches where microbial cultures were initially allowed to accumulate acids or metabolites before exposure to the mineral substrate, produced faster and more consistent REE release than direct inoculation systems. Mixed cultures of fungi and bacteria also demonstrated synergistic effects, with fungal acid production, complementing bacterial siderophore generation or redox activity. These synergisms highlight the potential for engineered consortia or synthetic microbial communities as next-generation bioleaching systems [43, 50, 53, 56, 59].

These findings illustrate that culture conditions serve as powerful biotechnological levers for enhancing REE bioleaching outcomes. Optimizing medium composition, pH trajectory, substrate loading, and process mode can dramatically alter extraction yields, often to a greater extent than can be achieved through microbial species selection alone. These parameters therefore represent critical components for developing scalable, high performance bioprocesses for REE recovery.

### Influence of mineral substrate properties

The mineralogical characteristics of the phosphate substrates exerted a substantial influence on microbial bioleaching outcomes, often determining which biochemical mechanisms dominated and the extent to which REEs could be mobilized. The effectiveness of REE solubilization varied according to mineral structure, accessory phase composition, buffering capacity, and REE speciation.

Monazite bearing minerals consistently exhibited the highest responsiveness to microbial bioleaching, particularly in organic acid driven fungal systems. Monazite, a predominant REE phosphate mineral, contains REEs bound within structures that are susceptible to proton attack and complexation by multi dentate organic acids such as citrate, oxalate, and gluconate. Across the studies, *Aspergillus*, *Penicillium*, and *Paecilomyces* spp. achieved rapid decreases in pH and substantial REE release when incubated with monazite rich matrices. The relatively high release of REE citrate and REE oxalate complexes, combined with strong ligand exchange at the mineral surface, contributed to efficient structural destabilization and lattice breakdown. These observations reinforce monazite as a favorable substrate for biotechnological processing, particularly in fungal driven reactors.

Apatite and other phosphate rock matrices yielded more modest dissolution efficiencies under comparable biochemical conditions. These minerals commonly contain REEs as minor substituents rather than as primary structural constituents, and their calcium phosphate frameworks are more resistant to acidolysis. Studies involving apatite and agricultural phosphate rocks reported lower extraction percentages and slower dissolution rates even under strong acidogenic metabolism, indicating that mineralogical resilience plays a major limiting role. Dissolution of apatite required sustained acidification and high concentrations of chelating metabolites and the competition from abundant Ca^2+^ ions hindered effective REE complex formation. These findings highlight that apatite dominant substrates may require either prolonged incubation, higher acid production, or engineered microbial consortia to achieve meaningful REE recovery.

Substrate accessory minerals played an additional role by modulating chemical and biological reactions at the mineral surface. The presence of iron bearing phases, such as those found in some monazite sands and processing residues, influenced bacterial redox mechanisms and siderophore activity. In iron-rich matrices, *Acidithiobacillus* spp. enhanced dissolution by oxidizing Fe^2+^ to Fe^3+^, creating ferric species that chemically attack the mineral surface. Siderophore producing bacteria further modified iron speciation and surface reactivity, enhancing REE mobilization through ligand mediated destabilization of Fe-P structures. Accessory dolomite or calcite phases increased buffering capacity, neutralizing microbial acids, and reducing dissolution efficiency unless acidogenic pathways were sufficiently strong to overcome these effects. Apart, studies using Florida phosphate tailings and mixed industrial residues reported extraction levels in the 10–40% range, with the upper values typically associated with optimized conditions or mixed culture approaches [47]. Further, it was not that carbonate-rich substrates neutralized microbial acids, suppressing dissolution. Mineral buffering capacity and presence of accessory minerals further influenced performance by either enhancing mechanistic diversity or suppressing acid driven dissolution.

These findings demonstrate that mineral substrate properties constitute a critical factor in determining bioleaching performance. Iron-bearing matrices offer unique opportunities to leverage bacterial redox processes, while buffering minerals pose challenges, requiring careful medium and process design. Understanding these mineralogical influences is essential for constructing robust, substrate appropriate biotechnological workflows for REE recovery.

### Influence of sterile and non-sterile mineral systems

An underreported but potentially significant variable across the reviewed studies is whether mineral substrates were sterilized prior to bioleaching experiments. The majority of investigations were conducted under sterile laboratory conditions to isolate the activity of inoculated strains and ensure experimental reproducibility. However, a limited number of comparative studies evaluated REE mobilization in sterile versus non-sterile phosphate mineral systems. In these comparisons, higher REE release was observed in non-sterile monazite systems relative to sterilized systems [46, 48]. The presence of indigenous microbial communities appeared to influence dissolution behavior, suggesting that native heterotrophic microorganisms may contribute additional organic acids or metal-complexing metabolites that enhance REE mobilization. These findings indicate that microbial interactions within mixed communities can modify bioleaching performance relative to monoculture systems. Despite these observations, systematic evaluation of sterile versus non-sterile systems remains limited, and most studies do not explicitly report mineral sterilization procedures. As a result, direct comparisons across studies are constrained.

### Kinetic patterns of REE bioleaching

The data summarized in Table 2 indicate that microbial bioleaching of phosphate minerals generally follows a multiphasic dissolution pattern. Most studies describe an initial lag phase associated with microbial adaptation and the onset of metabolite production, followed by a phase of accelerated REE release. A plateau or decline phase is then frequently observed, likely reflecting depletion of reactive mineral surfaces and secondary processes such as biosorption or REE-oxalate precipitation [43, 46, 53, 57]. Fungal systems, including *Aspergillus*, *Paecilomyces*, and *Penicillium*, commonly reached peak REE concentrations within 7–30 days, consistent with sustained organic acid production and prolonged microbe mineral contact. In contrast, bacterial systems operating under acidic conditions often demonstrated faster early-stage REE release within 24–96 h, although overall recovery remained strongly influenced by pulp density, particle size, and leaching configuration [43, 44, 47, 57]. Direct-contact bioleaching systems generally exhibited more rapid initial dissolution compared with indirect (spent-media) approaches, likely due to localized chemical gradients and microbial attachment at the mineral surface. However, indirect systems achieved comparable total REE release when sufficient concentrations of organic acids accumulated [43, 46]. Despite consistent reporting of cumulative REE recovery, relatively few studies conducted formal kinetic modeling. Approaches for determination of reaction rate constants were rarely applied, limiting cross-study comparability and quantitative assessment of rate-limiting steps.

Table 2 synthesize key attributes from the reviewed studies, including microbial species, substrate type, culture conditions, dominant mechanisms, and reported REE mobilization performance. This comparative summary provides a consolidated resource for identifying patterns, benchmarking outcomes, and guiding future biotechnological process development.

**Table 2:** Summary of studies on microbial bioleaching of REEs from phosphate minerals

Study/Reference	Microorganism(s)	Substrate Type	Culture Conditions	Dominant Mechanism(s)	REE Mobilization / Key Outcomes
Brisson et al. 2016 [43]	*Aspergillus niger*	Monazite	Two-step; AMS medium; 25–28 °C; 6 days; 1% pulp density; AMS medium	Organic acid production (citric, gluconic, oxalic, succinic)	~ 86 mg/L REEs in 6 days
	*Paecilomyces* WE3-F		Two-step; AMS medium; 25–28 °C; 6 days; 1% pulp density; AMS medium	Organic acid production (acetic, gluconic, succinic)	Highest reported levels: ~112 mg L^− 1^ REEs in 6 days
	*Aspergillus terreus* ML3-1		Two-step; 25–28 °C; 6 days; 1% pulp density; AMS medium	Organic acid production (itaconic, succinic)	~ 101 mg L^− 1^ REEs in 6 days
Brisson et al. 2020 [55]	*Paecilomyces * *sp. strain WE3-F*		One-step; 28 °C;6 days; AMS medium	Metabolites	~ 42 mg/L REEs
Keekan et al. 2017 [44]	*Aspergillus niger*		One-step; 30 °C; 60 days; Bromfield media	Citric, oxalic acids	Early release: ~ 0.7–1.4 mg L^− 1^ Ce
Keekan and Jalondhara, 2015 [41]			One-step; 32 °C; 60 days; 1% pulp density; Bromfield media	Acidification; biosorption	Ce dominant leachate:1.419 mg/L at day 15
Castro et al. 2020 [54]	*Aspergillus niger*		One-step; 30 °C; 15 days; 1% pulp density; Potato dextrose broth medium	Oxalic and citric acid pathway	Moderate REE dissolution; time dependent; 0.97 mg/L at day 3 (La, Ce, Nd)
Castro et al. 2023 [58]	*Burkholderia thailandensis*		One-step; 30 °C; 30days; 1% pulp density; Nutrient broth medium	Rhamnolipids with citric and oxalic acid driven dissolution	~ 8 mg/L in day 15
Hassanien et al. 2014 [40]	*Aspergillus ficuum*		One-step; 30 °C; 9 days; 0.6% pulp density; Modified Czapek’s-Dox medium	Citric and oxalic acid production	High REE dissolution (75.4%) with low pulp density
	*Pseudomonas aeruginosa*		One-step; 35 °C; 8 days; 0.6% pulp density; Nutrient agar medium	2-ketogluconic production	High REE dissolution (63.5%) with low pulp density
Kamal et al. 2012 [39]	*Aspergillus niger*	Phosphate rock	One-step; 10–15 days; C’zapeks-dox Broth medium	Organic-acid solubilization	REE dissolution of ~ 11. % - ~ 30. %
	*Penicillium* sp.		One-step; 10 days; C’zapeks-dox Broth medium	Organic-acid solubilization	REE dissolution of ~ 15% - ~17%
	*Pseudomonas fluorescence*		One-step; 10 days; C’zapeks-dox Broth medium	Organic-acid solubilization	REE dissolution of ~ 37%
Kang et al. 2020 [56]	*Aspergillus niger*	Monazite	One-step; 25 °C; 35 days; 2% pulp density; MCD medium	Citric and oxalic acid production	~ 43 mg/L REE leached by the 3rd week
Kang et al. 2021 [53]			One-step; 25 °C; 35 days; 2% pulp density; MCD medium	Citric and oxalic acid production	~ 1 mg/L REE leached by 4th week
Somasundaran et al. 2018 [47]	*Acidithiobacillus ferrooxidans*	Florida phosphate tailing	One-step; 30 °C; 2–3 days; 1% pulp density; 9 K medium	Acidic oxidation and complexation (sulfuric acid)	70% REE leached
	*Aspergillus niger*		One-step; 30 °C; 1 days; 1% pulp density; Modified Czapek’s- Dox liquid media	Citric and oxalic acid production	~ 42% REE leached
Corbett et al. 2017 [45]	*Penicillium* sp.	Monazite	One-step; 30–37 °C; 8 days; 0.5% pulp density; modified PVK media	Organic acid production (gluconic, citric, acetic)	12.32 mg/L REE leached from 192 h
Corbett et al. 2017 [46]		Sterile Monazite	One-step; 30–37 °C; 8 days; 0.5% pulp density; modified PVK media	Organic acid production	12.32 mg/L REE leached
Corbett et al. 2018 [48]		Non - Sterile Monazite	One-step; 30–37 °C; 8 days; 0.5% pulp density; modified PVK media	Organic acid production	23.7 mg/L REE leached
Corbett et al. 2018 [48]	*Penicillium* sp. CF1	Sterile Monazite	One-step; 30–37 °C; 14 days; 0.5% pulp density; modified PVK media	Organic acid production (oxalic, citric and phosphatases)	12.32 mg/L REE leached at day 8
		Non - Sterile Monazite	One-step; 30–37 °C; 14 days; 0.5% pulp density; modified PVK media	Organic acid production (oxalic, citric and phosphatases)	23.7 mg/L REE leached at day 08
Corbett et al., 2024 [65]	*Klebsiella aerogenes*	Monazite	One-step; 30 °C; 7 days; 0.5% pulp density; Modified NBRIP media	Organic-acid complexation (D-gluconic, oxalic, formic, acetic, alpha ketoglutaric, DL-malic and succinic)	REE dissolution of ~ 24mM
Fathollahzadeh et al. 2018 [49]	*Enterobacter aerogenes*	Monazite	One-step; 30 °C; 12 days; 1% pulp density; NBRIP medium	Organic-acid production	5.84 mg/L REE leached at day 12
Fathollahzadeh et al. 2018 [50]	Co culture *(Enterobacter aerogenes + Acidithiobacillus ferrooxidans)*		One-step; 30 °C; 12 days; 1% pulp density; Modified NBRIP media	Acidification and chelation	40 mg/L REE leached at day 09
Fathollahzadeh et al. 2019 [51]	*Enterobacter aerogenes*		One-step; 30 °C; 12 days; 1% pulp density; NBRIP medium, with glycine	Organic acid production	4 mg/L REE leached in MWM
Fathollahzadeh et al. 2019 [51]	*Acidithiobacillus ferrooxidans*		One-step; 30 °C; 12 days; 1% pulp density; Basal salt media, with glycine	Acidification and complexation	87 mg/L REE leached in MWM, at day 12 incubation
Nancucheo et al. 2019 [52]	*Acidithiobacillus ferrooxidans*		One-step; 30 °C; 30 days; Basal salts with trace elements liquid medium	Proton- and ferric-assisted dissolution	REE dissolution of ~ 5% - ~9%
	*Acidithiobacillus thiooxidans*		One-step; 30 °C; 30 days; Basal salts with trace elements liquid medium	Sulfuric acid dissolution	REE dissolution of ~ 2% - ~3%
Tian et al. 2022 [57]	*Acidithiobacillus ferrooxidans*	Phosphate rock	One-step; 30 °C; 14 days; 1% pulp density; 9k liquid medium	Fe²⁺ oxidation; EPS involvement	REE dissolution of ~ 28% (0.2025 mg/g)
Osman et al., 2019 [64]	*Aspergillus niger*	Phosphorites	One-step; 30 °C; 14 days; 1% pulp density; Siderophore in CAS assay	Siderophores involvement	REE dissolution of ~ 50% - ~66%
Shin et al. 2015 [42]	*Acetobacter aceti*	Monazite	One-step; 30 °C; 9 days; Reyes minimal medium	Organic-acid production (citric, malic, tartaric, acetic)	REE dissolution of ~ 2% - ~5%
Feng et al., 2011 [66]	*Pantoea agglomerans*	Fluorapatite	One-step; 25 °C; 26 days; Minimal medium	Organic-acid production	Measurable REE released
	*Bacillus megaterium*				
Bayarsaikhan et al., 2025 [59]	*Thiobacillus denitrificans* and *Bacillus cereus* mix culture	Phosphate rock	One-step; 7 days; 0.5% pulp density; 9k liquid medium	Organic-acid leaching	REE dissolution of 15.85%- Lugiin Gol (LG) deposit & 6.23%- Mushgia Khudag (MK) deposit
Bayarsaikhan et al., 2025 [60]	*Thiobacillus denitrificans*		One-step; 30 °C; 7 days; 9k liquid medium	Acidification + chelation	REE dissolution of 838.68 mg L^− 1^(2.4%)
	*Bacillus cereus*				REE dissolution of 1.12mg L^− 1^(0.003%)

## Discussion

Across the twenty-five studies included in this review, REE bioleaching performance exhibited substantial variability, reflecting differences in microbial species, mineral substrates, experimental conditions, and reporting formats. Although the reviewed studies collectively demonstrate proof-of-concept feasibility, methodological heterogeneity and inconsistent reporting frameworks limit direct quantitative comparison. Variations in pulp density, particle size, incubation duration, analytical detection limits, and reporting units introduce substantial uncertainty when benchmarking performance. Conclusions regarding relative microbial superiority must be interpreted cautiously and within experimental context. Standardized reporting metrics would significantly improve reproducibility and facilitate future meta-analytical assessments. Much of the available literature remains exploratory rather than mechanistically resolved. Many studies focus primarily on demonstrating REE release feasibility, with limited interrogation of rate-limiting steps, mineral surface transformations, or metabolite flux dynamics. As a result, reported efficiencies often lack sufficient mechanistic context to enable predictive comparison or rational process optimization across phosphate mineral systems.

Across the studies summarized in Table 2, the wide variation in reported REE recovery reflects not intrinsic superiority of any single microbial group, but the interaction between microbial metabolism, mineralogy, and process design. Fungal systems frequently achieved high cumulative REE release under low pulp density and extended incubation, due to sustained organic acid flux and prolonged microbe mineral contact. However, elevated dissolution did not always translate into high recoverable REE concentrations, as secondary precipitation and biomass-associated sorption reduced soluble fractions. Bacterial systems generally exhibited faster early-stage mobilization under acidic conditions, but were more sensitive to pulp density, mineral buffering capacity, and solution chemistry. Direct-contact systems consistently accelerated early kinetics compared to spent-medium approaches, although comparable overall recoveries were possible when organic acid concentrations were sufficiently high. Monazite-rich and fine-grained substrates showed greater responsiveness to bioleaching than apatite-dominant or highly crystalline concentrates, reinforcing the importance of mineral texture and surface accessibility. Two-step strategies and moderate pulp densities emerged as recurrent determinants of improved performance. These findings indicate that variability in REE bioleaching outcomes is governed more by process-substrate alignment than by taxonomic identity alone. A direct comparison of headline recovery values across studies can be misleading without contextualizing mineral characteristics and operational parameters. Standardized testing frameworks and integrated dissolution recovery assessments are therefore essential for realistic evaluation of industrial potential.

The studies illustrate that microorganisms can extract REE from phosphate minerals via a synergy of acidogenic, chelating, and redox-driven mechanisms. However, the effectiveness of these mechanisms varies substantially across microbial groups, mineral substrates, and culture conditions. The results underscore a key principle: REE bioleaching is governed not only by organism identity, but also by the alignment between microbial metabolism, medium chemistry, and mineralogical properties. A critical analysis of microbial performance reveals that fungi often outperform bacteria metabolically due to their superior capacity for high concentrations of multi-dentate organic acids production. This is likely driven by their efficient carbon flux pathways such as the tricarboxylic acid (TCA) cycle and gluconeogenesis, which enable high-yield secretion of chelating agents like citric and oxalic acids [81, 82]. These metabolites simultaneously lower pH and form strong complexes with REE^3+^ ions, resulting in rapid and direct attack of monazite and related phosphate lattices. The high performance of *Aspergillus*, *Penicillium*, and *Paecilomyces* reflects their metabolic flexibility, allowing them to divert carbon flux into acidogenesis under nutrient-limited or low pH circumstances. In contrast, bacteria rely more on proton extrusion and siderophore-mediated mechanisms, which are effective but typically yield lower dissolution rates owing to less intensive acidification and chelation in phosphate matrices [83, 84]. This metabolic advantage in fungi stems from their filamentous growth, which facilitates biofilm formation and localized acid gradients on mineral surfaces, enhancing lattice destabilization. This filamentous physiology also enables direct penetration into microfractures of mineral substrates, increasing mineral-biomass contact, and accelerating dissolution, compared to planktonic bacterial cells. However, direct cross-study comparison of fungal and bacterial performance must be interpreted cautiously. Reported high fungal efficiencies were frequently obtained at low pulp densities and extended incubation periods, conditions that may not directly translate to industrially relevant solids loading. Bacterial systems were often evaluated under shorter experimental durations or more acidic starting conditions. These differences in experimental design complicate definitive attribution of superiority to any single microbial group and highlight the need for standardized benchmarking protocols.

Emerging evidence from the reviewed literature indicates that biofilm-mediated mineral-microbe interactions can significantly influence REE bioleaching efficiency. Unlike purely solution-driven mechanisms, attached microbial populations generate localized microenvironments characterized by proton accumulation, EPS-associated ferric iron, and concentrated organic ligands at the mineral surface. Such interfacial conditions can accelerate phosphate matrix destabilization beyond what would be predicted by bulk chemical parameters. Comparative studies in which physical contact between microorganisms and phosphate minerals were either permitted or restricted, demonstrated consistently higher REE mobilization under contact-permitted conditions [49, 57]. Observations of dissolution rates exceeding equilibrium-based chemical predictions further support the contribution of surface-associated mechanisms [66]. These findings suggest that biofilm formation is not merely incidental but may represent a critical determinant of leaching kinetics and process efficiency. From a bioprocess engineering perspective, reactor configurations that promote controlled surface colonization could enhance sustained REE recovery while minimizing reagent input. Despite these observations, evaluation of biofilm architecture, quantitative characterization of biofilm thickness, EPS composition, or interfacial diffusion gradients and kinetics was rarely performed. Without such measurements, it remains difficult to distinguish the relative contributions of localized acidification, redox cycling, and purely chemical dissolution processes occurring within the biofilm matrix. This represents a key gap in translating surface-mediated mechanisms into controllable engineering parameters.

In addition to organic acid pathways, several studies also demonstrated the contribution of extracellular phosphatases, particularly in *Penicillium* spp. which catalyze hydrolysis of phosphate groups and complement acid-driven dissolution. This enzymatic mechanism was especially evident in strains such as *Penicillium* sp. CF1, where phosphatase activity synergized with chelation to enhance REE release. The parallel routes explain the accelerated dissolution found in co-culture and sequential processes. Across the studies, phosphatase activity was not consistently quantified, and in some cases enzymatic contributions were inferred indirectly from phosphate release data. The lack of standardized enzyme assays limits definitive assessment of the magnitude of enzymatic versus acid-driven dissolution pathways in REE mobilization [48].

The predominance of sterile laboratory systems in the reviewed literature contrasts with the inherently non-sterile nature of industrial phosphate ores and tailings. Indigenous microbial populations in non-sterile environments may influence bioleaching performance through cooperative metabolite production, competitive substrate utilization, or shifts in community composition. Enhanced REE mobilization observed in non-sterile systems suggests that community-level interactions may augment dissolution beyond single-strain activity. From a process engineering perspective, these findings have important implications. The potential to operate without strict sterilization could reduce operational costs and simplify scale-up. However, non-sterile conditions may also introduce variability, carbon source competition, or instability of engineered strains. The ecological robustness of selected bioleaching microorganisms under mixed-community conditions remains largely untested. Notably, very few studies systematically tracked microbial community shifts, metabolite profiles, or competitive exclusion dynamics in non-sterile systems. While enhanced REE mobilization has been observed in some non-sterile contexts, the reproducibility, and long-term stability of such community-driven effects remain uncertain. Controlled ecological studies will be essential to determine whether cooperative interactions can be reliably harnessed at scale. Future research should therefore incorporate controlled comparisons between sterile and non-sterile mineral systems to evaluate process resilience, kinetic stability, and scalability under conditions that better approximate industrial environments [46, 48].

While cumulative REE yields were frequently reported, systematic kinetic analysis were uncommon. The available evidence suggests that fungal systems often exhibit pronounced early solubilization phases coinciding with peak organic acid production, followed by slower extraction rates, potentially influenced by buffering effects or secondary mineral formation. Bacterial systems generally show more gradual dissolution profiles linked to progressive acidification or redox cycling. Across the studies, formal kinetic modeling approaches, such as shrinking-core models, diffusion-controlled frameworks, or determination of apparent rate constants were rarely applied. The absence of standardized kinetic descriptors restricts cross-study comparability and mechanistic interpretation and hinders rational scale-up design. Future investigations should combine time-resolved REE concentration data with mineral surface characterization to distinguish surface-reaction control from mass-transfer limitations. Incorporating quantitative rate analysis in future studies would strengthen process optimization and improve industrial relevance [43, 44, 46, 47, 53, 57].

Culture conditions emerged as equally crucial as the choice of microbe, medium composition, carbon-nitrogen balance, pH evolution, pulp density, and particle size all impacted metabolite synthesis and mineral accessibility. Pulp density strongly modulated microbial activity, with higher solids loading buffering acids and reducing dissolution efficiency, while excessively low pulp densities limited metabolite mineral interactions. The reviewed studies consistently showed optimal REE release at moderate pulp densities, where acid attack, microbial growth, and mineral accessibility were balanced. Particle size effects mirrored these constraints, although smaller particles increased reactive surface area, excessively fine fractions impaired oxygen transfer, caused cell damage, and intensified particle-particle collisions that disrupted microbial structures, ultimately reducing extraction. Several studies identified intermediate particle size ranges as optimal for sustaining both microbial physiology and mineral reactivity. Two-step bioleaching consistently increased REE release by enabling maximal metabolite buildup before mineral exposure. These data corroborate the notion that REE bioleaching should be seen as a bioprocess rather than a physiologically passive interaction. Most parameter optimization studies evaluated variables independently rather than through multivariate or factorial experimental designs. This fragmented approach limits understanding of interaction effects between temperature, pulp density, carbon source, and particle size. Integrated experimental frameworks would enable clearer identification of dominant constraints governing REE release efficiency.

However, the review’s findings must be interpreted in light of several limitations. The limited sample size of the studies restricts generalizability, potentially overlooking emerging microbial systems or substrates. Furthermore, potential publication bias, where studies with negative results (e.g., ineffective microbial strains or low REE yields) are underrepresented, may inflate perceived bioleaching efficacy. Additionally, the scarcity of systematic optimization studies on temperature, pulp density, and particle size, despite their demonstrated influence, limits mechanistic understanding of how these physical parameters constrain microbial activity and metabolite effectiveness. Gaps in the literature include limited exploration of economic feasibility, while bioleaching is generally more cost-effective and environmentally benign than chemical leaching with the lower operational costs due to reduced energy inputs and reagent use, versus higher upfront costs for chemical methods that generate hazardous wastes requiring expensive disposal. Even within the limited discussions, cost comparisons with conventional chemical leaching were largely qualitative. Few studies incorporated life-cycle assessment, reagent recycling analysis, or energy-balance calculations, leaving the true techno-economic competitiveness of microbial REE bioleaching insufficiently quantified. A persistent limitation across the literature also includes the use of shake-flask experiments and empirical parameter tuning. Few studies used contemporary biotechnology tools to quantify rate-limiting steps or analyses metabolic pathways. Because systems-level analysis is lacking, strain selection is primarily opportunistic rather than guided by metabolic design. This gap also applies to process development, although controlled bioreactor operation is crucial for scaling, reproducibility, and metabolite flux optimization, very few studies have tried it. Also, the reviewed studies predominantly feature bacteria and fungi, but, archaea and yeasts have received limited attention in REE bioleaching from phosphate minerals, likely due to their lower acid production compared to fungi or specialized bacterial mechanisms. However, emerging research on archaeal extremophiles suggests potential for integration in mixed consortia under harsh conditions, warranting further investigation [85]. Yeasts, though rarely studied, may also offer underexplored advantages such as robust tolerance to osmotic stress and the ability to secrete specialized organic acids under high-sugar media conditions, representing another gap in the current dataset. In addition, selective reporting of positive extraction outcomes may obscure instances of low-yield or unsuccessful microbial systems, potentially inflating perceived overall feasibility. Transparent reporting of negative or low-performance datasets would substantially strengthen the evidence base.

The next advances in microbial REE bioleaching will depend on moving beyond organism screening toward rational, biotechnology-enabled process development. For instance, CRISPR-Cas systems have been successfully applied in strain engineering for bioleaching, such as in *Acidithiobacillus ferrooxidans*, where CRISPR/dCas12a was used to knock down genes involved in sulfur metabolism, enhancing iron oxidation and metal recovery efficiency in sulfide ores, a strategy adaptable to REE phosphate bioleaching for improved acid production [86]. Similarly, CRISPR-Cas9 has enabled scarless genome editing in *Acidithiobacillus ferridurans* to optimize redox pathways, which could provide a pathway to increase siderophore secretion in bacterial consortia for REE mobilization [87].

Further advances in rare-earth biotechnology beyond direct phosphate mineral bioleaching expand the strategic design space for integrated recovery systems. In addition to native acidification capacity, engineered variants have been explored to enhance organic acid flux and metal solubilization efficiency. Studies from Reed et al. have demonstrated the capacity of *Gluconobacter oxydans* to mobilize rare earth elements through oxidative glucose metabolism and gluconic acid production [88–90]. Work from Barstow et al. has also demonstrated the engineering of *G.oxydans* to enhance oxidative glucose metabolism and increase organic acid flux for metal solubilization [91, 92]. By targeting periplasmic dehydrogenase systems and metabolic pathways linked to gluconic acid production, these engineered strains achieved improved metal release efficiencies in laboratory systems. Complementing these dissolution-focused strategies, studies from Park et al. have demonstrated that the lanthanide-binding protein lanmodulin (LanM), originally identified in *Methylobacterium extorquens*, exhibits exceptionally high selectivity and affinity for trivalent rare earth elements in aqueous environments [93–95]. Engineered LanM variants have achieved single-step separation of closely related lanthanides with high purity, and comparative screening of LanM homologs has revealed tunable selectivity across individual REEs. Protein-based binding systems are applied primarily to REE separation rather than mineral dissolution. They offer a biologically derived downstream recovery solution that could be integrated with microbial solubilization platforms. Although these studies were not limited to primary phosphate minerals, the mechanistic framework, oxidative acid generation coupled with controlled metabolite secretion has direct relevance for improving dissolution of phosphate-bound REEs. These findings illustrate the feasibility of rational strain engineering approaches to enhance acid production and mineral dissolution, coupled with selective biological ligands for post-leaching REE capture and purification.

**Fig. 5:**
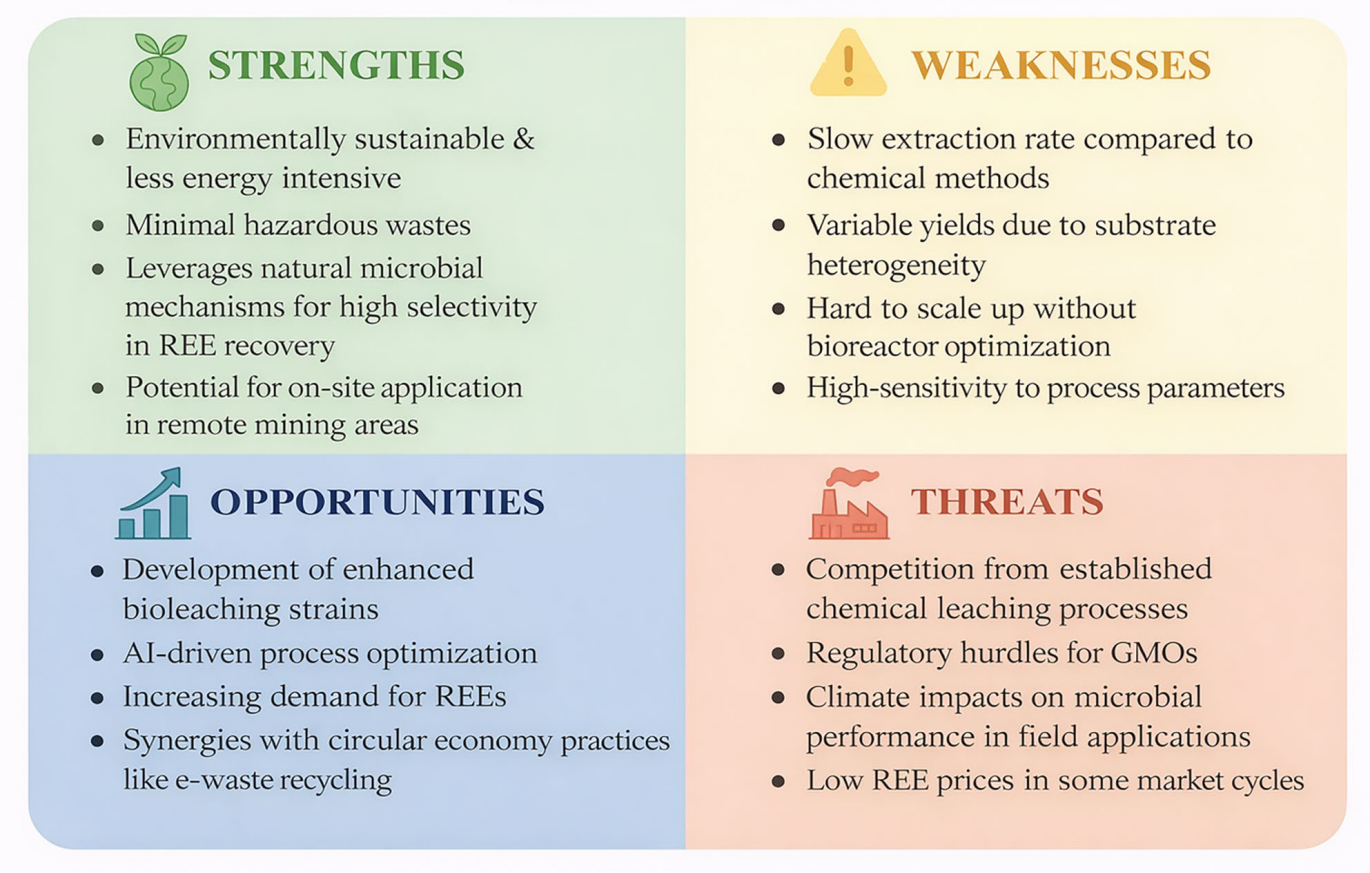
SWOT analysis of microbial bioleaching for the recovery of REEs from phosphate minerals to provide a strategic framework to guide future research and industrial translation

Also, AI-driven digital twins have simulated bioleaching processes for metal recovery, utilizing sensor data and predictive analytics to real-time adjust parameters like pH and temperature in real time, thereby achieving higher efficiency gains. Such insights could accelerate REE bioleaching scale-up through predictive modeling of fungal-bacterial synergies [96, 97]. Metabolic engineering and omics-driven pathway discovery represent another two particularly noteworthy strategic directions [98, 99]. Identifying regulatory bottlenecks in the synthesis of siderophores, redox metabolism, and organic acid production, requires integrated genomic, transcriptomic, proteomic, and metabolomic analyses. These discoveries would allow the targeted engineering of high-performing bacterial and fungal strains with improved chelator secretion, decreased metabolite reabsorption, and increased acidogenesis. Integrating these strain-engineering efforts, with data on optimal particle sizes, pulp densities, and temperature ranges, would enable the construction of microbial chassis specifically tuned to physical and chemical constraints imposed by phosphate ore types. Additionally, the complementary functions of iron-oxidizing bacteria, heterotrophic chelator producers, and fungal acid producers, could result in synthetic consortia that have stronger dissolution environments than individual organisms. A route to predictable consortium behaviour is provided by synthetic ecology tools such as communication modules, population control circuits, and metabolite flux balancing [100]. Moving to controlled reactors that control pH, oxygen, substrate feeding, and metabolite accumulation is necessary for scaling. While in situ product removal could reduce reprecipitation losses, fed-batch, semi-continuous, and continuous configurations may be able to overcome the drawbacks of batch systems. Further, prior to bioleaching, materials that are carbonate-buffered and rich in apatite will need to have their surfaces modified. Mechanochemical activation, microfracturing, and mild acid preconditioning are examples of low-intensity techniques that can boost the reactive surface area without compromising the process’s overall sustainability. These pretreatment strategies also mitigate challenges associated with fine particle effects, such as oxygen transfer limitations and microbial cell damage by increasing surface reactivity without producing excessively fine particulates. To assess practical viability, future research should use technical and life-cycle assessments, and pilot-scale testing. The operating windows during which microbial REE recovery is both economically and environmentally beneficial will be determined by early integration of these evaluations. With these developments, microbial bioleaching has the potential to develop from a viable laboratory idea into a strong industrial approach for the long-term recovery of rare earth elements. A strategic SWOT analysis highlighting the current status and future trajectory of microbial bioleaching as a sustainable technology for REE recovery is presented in Fig. 5. The framework identifies key internal advantages/disadvantages and external drivers that must be addressed to achieve industrial implementation.

## Conclusions

Microbial bioleaching offers a promising and environmentally compatible route for recovering rare earth elements from phosphate minerals. Filamentous fungi show the highest dissolution capacity through robust organic-acid production, while bacterial systems contribute valuable chelation and redox mechanisms that enhance performance in mixed cultures. The effectiveness of these microbial strategies, however, is strongly dependent on bioprocess parameters and mineralogical context. Across the reviewed studies, nearly all experiments were conducted at laboratory scale, with only a single bioreactor-based investigation reported, underscoring the early developmental stage of REE bioleaching technologies. Variability in growth media composition, carbon sources, and pH trajectories, and limited temperature-dependent datasets further highlight the methodological inconsistencies that currently prevent establishing definitive correlations between operating conditions and REE extraction efficiencies. Current evidence remains insufficient to define universally optimal operating windows due to heterogeneity in experimental design and reporting practices across studies. The absence of standardized kinetic descriptors and reactor-scale validation further limits extrapolation of laboratory findings to continuous processing environments. The current body of research establishes feasibility but remains largely empirical, with limited mechanistic resolution and minimal application of advanced biotechnology or controlled process engineering. Critical mechanisms including organic acid synthesis, phosphatase activity, bacterial adhesion, and phosphate regulation require deeper and organism-specific investigation to clarify their relative contributions to REE mobilization. To transition from laboratory demonstrations to practical implementation, future work must integrate systems biology, strain engineering, bioreactor optimization, substrate-specific process tailoring, quantitative kinetic modeling, interfacial biofilm characterization, and multivariate process optimization to establish reproducible performance benchmarks. Sustainable scale-up will also require addressing process-integrated challenges such as waste management, buffering effects of mineral matrices, and the development of economically viable, low-energy workflows that compete with conventional chemical leaching. Overall, microbial REE bioleaching represents a viable foundation for sustainable mineral processing, but its industrial potential will only be realized through a shift toward mechanistically informed, engineered, and scalable biotechnological workflows. The persistent lack of standardized protocols and limited insight into individual microbial contributions remain significant barriers to progress. Further targeted research is urgently needed to elucidate precise mechanisms and optimize consortia for industrial-scale REE bioleaching from diverse phosphate resources. At the current state microbial REE bioleaching should be regarded as a technologically promising, yet, developmentally immature platform that requires coordinated advances in mechanistic microbiology, mineral surface science, and process systems engineering. This biotechnological approach aligns directly with several United Nations Sustainable Development Goals (SDGs), including SDG 7 (Affordable and Clean Energy) by enabling REE supply for renewable technologies; SDG 9 (Industry, Innovation, and Infrastructure) through engineered microbial systems; SDG 12 (Responsible Consumption and Production) via reduced hazardous waste compared to chemical methods; and SDG 13 (Climate Action) by lowering the carbon footprint of extraction processes.

## Supplementary Information

Below is the link to the electronic supplementary material.

Supplementary Material 1

## Data Availability

The datasets supporting the conclusions of this article are included within the article and as Supplementary Materials.
