# Supplementary material for: Microbial bioleaching of rare earth elements from phosphate minerals: a biotechnology-driven systematic review of mechanisms, bioprocess determinants, and opportunities for sustainable recovery
Source: BMC Biotechnol. 2026 Mar 3;26:40. doi: 10.1186/s12896-026-01125-1 (PMC13064222; doi:10.1186/s12896-026-01125-1)
Supplement: Supplementary file 1 — Supplementary Material 1 [file 12896_2026_1125_MOESM1_ESM.docx]

**Microbial Bioleaching of Rare Earth Elements from Phosphate Minerals: A Biotechnology-Driven Systematic Review of Mechanisms, Bioprocess Determinants, and Opportunities for Sustainable Recovery**

Sonali Prabodha Vijayarathna^1^, Ileperumaarachchige Vayanga Nishani Rathnayake^2^, Pradeep Wishwanath Samarasekere^1^*

1. Center for Advanced Materials and Smart Manufacturing, University of Kelaniya, Kelaniya 11600, Sri Lanka
2. Department of Microbiology, Faculty of Science, University of Kelaniya, Kelaniya 11600, Sri Lanka

* Corresponding author

Pradeep Wishwanath Samarasekere

[pradeep.samarasekere@gmail.com](mailto:pradeep.samarasekere@gmail.com)

# Supplementary Tables

Table S 1. The most effective microorganisms identified in comparative studies in bioleaching process of REE

| **Microorganisms Used in the Study** | **Most effective microorganism** | **Reference** |
| --- | --- | --- |
| *Aspergillus niger, Aspergillus terreus* strain ML3-1, *Paecilomyces* sp. strain WE3-F | *Paecilomyces* sp. strain WE3-F | (43) |
| *Penicillium sp, Aspergillus niger, Aspergillus tubigensis, Pantoea agglomerans, Enterobacter aerogenes, Pseudomonas aeruginosa, Pseudomonas putida, Klebsiella pneumoniae, Klebsiella oxytoca, Bacillus megaterium* | *Penicillium* sp. | (45) |
| *Penicillium* sp.*, Enterobacter aerogenes, Pantoea agglomerans, Pseudomonas putida* | *Penicillium* sp. | (46) |
| *Penicillium* sp.CF1, *Enterobacter aerogenes, Pantoea agglomerans, Pseudomonas putida* | *Penicillium* sp.*CF1* | (48) |
| *Klebsiella aerogenes,* *Pseudomonas putida, Gluconobacter oxydans, Burkholderia T48* | *Klebsiella aerogenes* | (65) |
| *Azospirillum brasilense, Mesorhizobium cicero, Azospirillum lipoferum, Pseudomonas rhizosphaerae, Acetobacter aceti* | *Acetobacter aceti* | (42) |
| *Thiobacillus denitrificans, Bacillus cereus* | *Thiobacillus denitrificans* | (60) |

Table S 2. Summary of the culture mediums used for bioleaching

| **Source** | **Microorganism used for leaching** | **Culture medium** | **Reference** |
| --- | --- | --- | --- |
| Monazite | *Aspergillus niger* | 1. Bromfield media (BM) [the most effective medium] 2. Sucrose media (SM) | (41) |
|  | *Aspergillus niger* | 1. Bromfield media (BM) [the most effective medium] 2. Sucrose media (SM) 3. Synthetic media (SynM) 4. Standard media (StdM) | (44) |
|  | *Aspergillus niger* | Potato dextrose broth | (54) |
|  | *Aspergillus niger*  *Aspergillus terreus strain ML3-1*  *Paecilomyces sp. strain WE3-F* | 1. NBRIP medium 2. Modified Pikovskaya medium 3. Pikovskaya medium without Mn and Fe 4. Modified ammonium salts medium (AMS medium) | (43) |
|  | *Paecilomyces sp. strain WE3-F* | Modified ammonium salts medium (AMS medium) | (55) |
|  | *Aspergillus niger* | MCD medium | (56) |
|  | *Aspergillus niger* | MCD medium | (53) |
|  | *Penicillium sp* | PVK medium | (45) |
|  | *Enterobacter aerogenes* | National Botanical Research Institute Phosphate (NBRIP) medium | (50) |
|  | *Acidithiobacillus ferrooxidans* | Basal salt media (BSM) |  |
|  | Co culture *Enterobacter aerogenes* and  *Acidithiobacillus ferrooxidans* | Modified NBRIP media |  |
|  | *Enterobacter aerogenes* | National Botanical Research  Institute Phosphate (NBRIP) medium | (51) |
|  | *Acidithiobacillus ferrooxidans* | Basal salt media (BSM) |  |
|  | *Acidithiobacillus ferrooxidans* | Basal salts with trace elements liquid medium | (52) |
|  | *Acidithiobacillus thiooxidans* | Basal salts with trace elements liquid medium |  |
|  | *Acetobacter aceti* | Reyes minimal medium | (42) |
|  | *Aspergillus ficuum* | Modified Czapek's-Dox agar (MCDA) medium | (40) |
|  | *Pseudomonas aeruginosa* | Nutrient agar (NA) |  |
|  | *Klebsiella aerogenes* | Modified National Botanical Research Institute's Phosphate medium with fructose | (65) |
|  | *Burkholderia thailandensis* | Nutrient broth medium | (54) |
| Sterile Monazite | *Penicillium sp* | modified PVK media | (45) |
|  | *Penicillium sp.CF1* | modified PVK media | (48) |
| Non - Sterile Monazite | *Penicillium sp* | modified PVK media | (45) |
|  | *Penicillium sp.CF1* | modified PVK media | (48) |
| Florida phosphate tailing | *Acidithiobacillus ferrooxidans* | 9K medium | (47) |
|  | *Aspergillus niger* | Modified Czapek’s- Dox liquid media |  |
| Phosphate rock | *Acidithiobacillus ferrooxidans* | 9k liquid medium | (57) |
|  | *Aspergillus niger* | C’zapeks-dox Broth medium | (39) |
|  | *Penicillium sp.* |  |  |
|  | *Pseudomonas fluorescence* |  |  |
|  | *Thiobacillus denitrificans* | 9k liquid medium | (59) (60) |
|  | *Bacillus cereus* |  |  |
| Fluorapatite | *Pantoea agglomerans* | Minimal medium containing 0.012 g/L KBr, 0.2 g/L glucose, and 0.38 g/L NH4Cl and fluorapatite | (66) |
|  | *Bacillus megaterium* |  |  |
| Phosphorites | *Aspergillus niger* | Siderophore in CAS assay | (64) |

Table S 3. Summary of bioleaching studies from primary REE resources (n.m-not mentioned)

| **Microorganism used for leaching** | **Source** | **Extracted REE** | **Leaching agents** | **Pulp density (% w/v)** | **Temp (°C)** | **Time (d)** | **Total REE leaching efficiency (%)** | **Total REE leaching efficiency** | **Reference** |
| --- | --- | --- | --- | --- | --- | --- | --- | --- | --- |
| *Aspergillus niger* | Monazite | Ce | n.m | 1 | 32 | 60 | n.m | 1.419 mg/L at day 15 | (41) |
|  |  | Ce | oxalic, acetic, citric, succinic, kojic, itaconic | 2 | 30 | 60 | Ce (100) | 0.701 mg/L in Bromfield  Media at day 30 | (44) |
|  |  | La, Ce, Nd | Citric  Gluconic | 1 | 30 | 15 | n.m | 0.97 mg/L at day 3 | (54) |
|  |  | La, Ce, Nd, Pr, | Citric, Gluconic, Oxalic, Succinic | 1 | 25 - 28 | 6 | n.m | 86 ± 6 mg/L | (43) |
|  |  | La, Ce | citric, oxalic | 0.5 – 2 | 25 | 35 | n.m | 43.1 mg/L by the 3^rd^ week | (56) |
|  |  | Ce | citric, oxalic | 2 | 25 | 35 | n.m | ~1 mg/L by 4^th^ week | (53) |
|  | Florida phosphate tailing | n.m. | Citric, oxalic | 1 | 30 | 1 | 42.5 % | n.m | (47) |
|  | Phosphate rock | n.m. | n.m. | n.m. | n.m. | 10 - 15 | 11.4 %  30.2 % | n.m | (39) |
|  | Phosphorites | Sm,  La, Ce | Siderophores | 1 | 30 | 2 | Sm 66.7%,  La 51%, Ce 50.1% | n.m. | (64) |
| *Aspergillus terreus strain ML3-1* | Monazite | La, Ce, Nd, Pr, | itaconic, succinic | 1 | 25 - 28 | 6 | n.m. | 101 ± 27 mg/L | (43) |
| *Paecilomyces sp. strain WE3-F* |  | La, Ce, Nd, Pr, | acetic, gluconic, succinic | 1 | 25 - 28 | 6 | n.m. | 112 ± 16 mg/L | (43) |
|  |  | REE | 210 metabolites | n.m | 28 | 6 | n.m | 42 ± 15 mg/L | (55) |
| *Aspergillus ficuum* |  | n.m | citric, oxalic | 0.6 | 30 | 9 | 75.4 % | n.m | (40) |
| *Penicillium sp* |  | La, Ce, Nd, Pr | Gluconic  Citric  Acetic | 0.5 | 30-37 | 8 | n.m | 12.32 mg/L from 192hr | (45) |
|  | Sterile Monazite | La, Ce, Nd, Pr | n.m | 0.5 | 30-37 | 8 | n.m | 12.32 mg/L^1^ | (46) |
|  | Non - Sterile Monazite | La, Ce, Nd, Pr | n.m | 0.5 | 30-37 | 8 | n.m | 23.7 mg/L | (46) |
|  | Phosphate rock | n.m | n.m | n.m | n.m | 10 | 15.9% 17.8% | n.m. | (39) |
| *Penicillium sp.CF1* | Sterile Monazite | La, Ce, Nd, Pr | oxalic, citric, phosphatases | 0.5 | 30-37 | 14 | n.m | 12.32 mg/L at day 8 | (48) |
|  | Non - Sterile Monazite | La, Ce, Nd, Pr | oxalic, citric, phosphatases | 0.5 | 30-37 | 14 | n.m. | 23.7 mg/L day 08 | (48) |
| *Penicillium sp.CF1* | Sterile Monazite | La, Ce, Nd, Pr | oxalic, citric, phosphatases | 0.5 | 30-37 | 14 | n.m. | 12.32 mg/L at day 8 | (48) |
| *Enterobacter aerogenes* | Monazite | La, Ce, Nd, Pr, Y | n.m. | 1 | 30 | 12 | n.m. | 5.84 mg/L at day 12 | (49) |
|  |  | La, Ce, Nd, Pr, Y | n.m. | 1 | 30 | 12 | n.m. | 4 mg/L in MWM | (51) |
| *Acidithiobacillus ferrooxidans* | Monazite | La, Ce, Pr, Nd | n.m | 1 | 30 | 12 | n.m | 87 mg/L, at day 12 incubation |  |
|  |  | La, Ce | n.m | n.m | 30 | 30 | ~9 % Ce  ~5% La | n.m | (52) |
|  | Florida phosphate tailing | n.m. | sulfuric acid | 1 | 30 | 2-3 | 70 % | n.m | (47) |
|  | Phosphate rock | Ce, La, Nd, Y, | n.m | 1 | 30 | 14 | 28.46 % | 0.2025 mg/g | (57) |
| Co culture *(Enterobacter aerogenes + Acidithiobacillus ferrooxidans)* | Monazite | La, Ce, Nd, Pr, Y | n.m | 1 | 30 | 12 | n.m | 40 mg/L at  day 09 | (50) |
| *Pseudomonas aeruginosa* |  | n.m | 2-ketogluconic | 0.6 | 35 | 8 | 63.5 % | n.m | (40) |
| *Acidithiobacillus thiooxidans* |  | La, Ce | Sulfuric acid | n.m | 30 | 30 | ~3 % Ce  ~2 % La | n.m | (52) |
| *Pseudomonas fluorescence* | Phosphate rock | n.m. | n.m. | n.m. | n.m. | 10 | 37.6 % | n.m | (39) |
| *Acetobacter aceti* | Monazite | La, Ce, Nd, Pr | citric, malic, tartaric, acetic | 16.7 | 30 | 9 | 0.13 % Ce  0.11 % La | ~5.7 mg/L Ce & 2.8 mg/L La | (42) |
| *Pantoea agglomerans* | Fluorapatite | La,Ce,Pr,Nd,  Sm,Eu,Gd,  Tb,Dy,Ho,Er,Tm,Yb,Lu | n.m. | 0.006 | 25 | 26 | n.m. | n.m. | (66) |
| *Bacillus megaterium* |  |  |  |  |  |  |  |  |  |
| *Thiobacillus denitrificans* | Phosphate rock | REE | n.m. | 50 | 30 | 7 | 2.4% | 838.68 mg L^−1^ | (60) |
| *Bacillus cereus* |  | REE | n.m. | 50 | 30 | 7 | 0.003% | 1.12mg L^−1^ |  |
| *Thiobacillus denitrificans* and  *Bacillus cereus* mix culture |  | REE | n.m. | 0.5 | n.m | 7 | 15.85%- Lugiin Gol (LG) deposit  6.23%- Mushgia Khudag (MK) deposit | n.m. | (59) |
| *Klebsiella aerogenes* | Monazite | REE | D-gluconic, oxalic,  formic, acetic, alpha ketoglutaric, DL-malic and succinic | 0.5 | 30 | 7 | n.m. | 24.03 ± 0.1 mM | (65) |
| *Burkholderia thailandensis* | Monazite | REE | Rhamnolipids | 1 | 30 | 21 | n.m. | 8.25 ± 0.04 mg/L (in day 15) | (58) |
